# The management of non-valvular atrial fibrillation (NVAF) in Australian general practice: bridging the evidence-practice gap. A national, representative postal survey

**DOI:** 10.1186/1471-2296-9-62

**Published:** 2008-11-13

**Authors:** Melina Gattellari, John M Worthington, Nicholas A Zwar, Sandy Middleton

**Affiliations:** 1 School of Public Health and Community Medicine, The University of New South Wales and Centre for Research Management, Evidence and Surveillance Sydney South West Area Health Service, Locked Bag 7008, Liverpool, NSW 1871, Australia; 2 Department of Neurophysiology, Sydney South West Area Health Service & Stroke and Neurology Services, Northern Beaches Hospitals, Locked Bag 7017, Liverpool, NSW 1871, Australia; 3 School of Public Health and Community Medicine. The University of New South Wales Sydney, NSW 2052, Australia; 4 Nursing Research Unit, St Vincents and Mater Health Sydney & National Centre for Clinical Outcomes Research (NaCCOR), Nursing and Midwifery, ACU National, PO Box 968, North Sydney, NSW 2059, Australia

## Abstract

**Background:**

General practitioners (GPs) are ideally placed to bridge the widely noted evidence-practice gap between current management of NVAF and the need to increase anticoagulant use to reduce the risk of fatal and disabling stroke in NVAF. We aimed to identify gaps in current care, and asked GPs to identify potentially useful strategies to overcome barriers to best practice.

**Methods:**

We obtained contact details for a random sample of 1000 GPs from a national commercial data-base. Randomly selected GPs were mailed a questionnaire after an advance letter. Standardised reminders were administered to enhance response rates. As part of a larger survey assessing GP management of NVAF, we included questions to explore GPs' risk assessment, estimates of stroke risk and GPs' perceptions of the risks and benefits of anticoagulation with warfarin. In addition, we explored GPs' perceived barriers to the wider uptake of anticoagulation, quality control of anticoagulation and their assessment of strategies to assist in managing NVAF.

**Results:**

596 out of 924 eligible GPs responded (64.4% response rate). The majority of GPs recognised that the benefits of warfarin outweighed the risks for three case scenarios in which warfarin is recommended according to Australian guidelines. In response to a hypothetical case scenario describing a patient with a supratherapeutic INR level of 5, 41.4% of the 596 GPs (n = 247) and 22.0% (n = 131) would be "highly likely" or "likely", respectively, to cease warfarin therapy and resume at a lower dose when INR levels are within therapeutic range. Only 27.9% (n = 166/596) would reassess the patient's INR levels within one day of recording the supratherapeutic INR. Patient contraindications to warfarin was reported to "usually" or "always" apply to the patients of 40.6% (n = 242/596) of GPs when considering whether or not to prescribe warfarin. Patient refusal to take warfarin "usually" or "always" applied to the patients of 22.3% (n = 133/596) of GPs. When asked to indicate the usefulness of strategies to assist in managing NVAF, the majority of GPs (89.1%, n = 531/596) reported that they would find patient educational resources outlining the benefits and risks of available treatments "quite useful" or "very useful". Just under two-thirds (65.2%; n = 389/596) reported that they would find point of care INR testing "quite" or "very" useful. An outreach specialist service and training to enable GPs to practice stroke medicine as a special interest were also considered to be "quite" or "very useful" by 61.9% (n = 369/596) GPs.

**Conclusion:**

This survey identified gaps, based on GP self-report, in the current care of NVAF. GPs themselves have provided guidance on the selection of implementation strategies to bridge these gaps. These results may inform future initiatives designed to reduce the risk of fatal and disabling stroke in NVAF.

## Background

Non-valvular atrial fibrillation (NVAF) is a common heart arrhythmia, affecting one in 20 people over the age of 65 and one in ten over the age of 75 [1]. NVAF is a significant risk factor for fatal and disabling ischaemic stroke [1] and is implicated in about 15% of all ischaemic strokes and as many as 30% of strokes occurring in people in their 80's [2].

Level I evidence demonstrates that anticoagulation with warfarin reduces the risk of stroke by 64% in relative terms when compared with placebo [3] and by 39% when compared with antiplatelets [4]. The absolute reduction in stroke risk attributable to warfarin highlights the effectiveness of anticoagulation, although the risk remains elevated even with treatment. The annual risk of stroke for people with NVAF at "high risk" ranges from 8.5% to 18.2% [5]. With warfarin, this risk can be reduced to approximately 3% to 6.6% per year. Consequently, international guidelines, such as those conjointly published by the American College of Cardiology, the American Heart Association and the European Society of Cardiology [6], recommend warfarin for NVAF, particularly for patients who have more than one moderate risk factor (eg congestive heart failure, hypertension, age greater than 75 years) or who have "high risk" factors (previous stroke, TIA or embolism).

Previous randomised controlled trials of anticoagulation with warfarin, however, were limited as these were carried out in specialist, tertiary centres, often recruiting younger patients than those typically encountered in the primary health care setting [3]. However, the recently published Birmingham Atrial Fibrillation Treatment of the Aged (*BAFTA*) trial demonstrated the effectiveness of warfarin in routine clinical practice amongst an elderly cohort (average 81.5 years) recruited and managed by GPs [7]. The results also indicated that warfarin was just as safe as aspirin.

Opportunities for reducing stroke risk in people with NVAF are frequently missed [8-10]. The National Institute of Clinical Studies of Australia has summarised the large, unrealised benefit of anticoagulation, stating: *"For every 1000 people with atrial fibrillation given anticoagulants, 25 will avoid experiencing a stroke if they take oral anticoagulants and 12 will avoid dying from a stroke" *[11].

In general, implementation of evidence in practice is often slow and incomplete. Knowledge gaps, organisational constraints, cognitive biases during decision-making and conservative attitudes towards behavioural change in response to "new" evidence may prevent or attenuate translation of evidence into practice [12]. There are specific barriers to the wider uptake of warfarin. Initiating and maintaining patients on warfarin is a complex task which presents several barriers for both GPs and patients. First, the risk of stroke must be assessed to identify patients in greatest need of anticoagulation [1,6]. Second, the selection of appropriate management requires rational consideration of the benefits and risks of treatment. Third, effective therapeutic use of anticoagulation requires regular blood monitoring of the international normalised ratio (INR) and dose titration to maintain therapeutic levels (2.0–3.0) [13]. Regular monitoring of INR levels is required as foods rich in vitamin K and certain drugs interact with the anticoagulant effect of warfarin. GPs must therefore ensure patients are tested regularly and have mechanisms in place for providing timely feedback of results and immediate dosage titration when required. Such requirements necessitate organisational systems for patient recall as well as effective communication between GPs and patients to ensure patients understand the importance of attending for regular monitoring. Fourth, these and other barriers to the wider implementation of warfarin need to be overcome and strategies to facilitate implementation must be feasible and acceptable to GPs.

The management of atrial fibrillation remains sub-optimal almost ten years since the publication of the first meta-analysis evaluating the effectiveness of warfarin [3]. The current literature suggests that simply improving physician knowledge and raising awareness of the evidence would be insufficient to remedy the evidence-practice gap. Fears about the side-effects of warfarin appear to affect physician prescribing. It has been shown that doctors reduce their prescribing of warfarin after one of their patients with NVAF has been hospitalised for a major bleeding event. However, rates of warfarin prescribing remain unchanged after a patient with NVAF experiences a stroke [10]. As discussed by others [10], these findings suggest that physicians are uncomfortable with making decisions that could actively induce harm. This contrasts to committing "acts of omission" where adverse outcomes, such as stroke, are less likely to be attributed to the actions of a physician.

Patient factors are also relevant. In a review of eight studies assessing patient preferences [14], six studies reported that fewer patients favoured warfarin compared with what would have been expected if evidence-based recommendations had been followed. However, results also indicate patient willingness to take warfarin. In one study comparing physician and patient preferences, patients had a lower threshold for accepting warfarin, with patients reporting that warfarin use is justified if 1.8 strokes in 100 people were avoided over two years [14]. By comparison, physicians indicated that an average of 2.5 strokes would need to be avoided over two years to justify warfarin. Recent evidence indicates that patient preferences may be affected by biases about warfarin which may not be "rational". In a study evaluating a patient decision tool, a significantly greater proportion of patients endorsed warfarin when "blinded" to the drug name compared to when the drug name was disclosed [15]. Qualitative research with physicians also reports patient aversion to using warfarin due to negative connotations [16].

To our knowledge, GPs have rarely been engaged in the identification of barriers to managing NVAF, nor have they been invited to comment on potentially useful strategies to overcome barriers. Existing support for GPs to appropriately manage NVAF and stroke risk has not been assessed. As part of a larger national survey [17], we assessed GPs' perceptions about the risks and benefits of warfarin and the services available to them to better manage NVAF. We sought GPs' views on strategies for better managing NVAF.

## Methods

### Recruitment

The contact details of 1000 Australian GPs were randomly selected from a commercial database [18]. The company housing the commercial data-base reports wide coverage and provides updated contact details. According to the most recently available information (September 2008), the commercial data-base had 22,669 doctors listed as GPs [18]. The data-base sources contact details from, for example, state medical registers, professional college and society lists, postgraduate councils, and a national directory of medical practitioners in Australia. The Australian Government Department of Health and Ageing listed 25,564 GPs in Australia in September 2007 [19]. However, the Department cautions that their headcount figures will overstate the number of active GPs [19]. It is our understanding that the Australian Government does not release mailing addresses of GPs to academic researchers, preferring instead to carry out mail-outs "in-house". We therefore opted for a list of GPs with updated contact details from which we could directly contact the participants of the survey and administer reminders to improve response rates.

Randomly selected GPs were mailed a questionnaire after an advance letter. The survey was carried out from October 2005 until July 2006. Demographic and practice characteristics have been reported elsewhere [17] and are shown in Table 1 (additional file 1). Post code of practice enabled us to determine the extent of socio-economic disadvantage of the area in which GPs served using a standardised index of relative socioeconomic disadvantage (IRSD) widely implemented in Australian government statistics and research [20]. The index of disadvantage assigns decile scores ranking suburbs from the most (score of 1) to the least socio-economic disadvantaged (score of 10). The score takes into account the prevalence of low income, low educational attainment and unemployment [20].

### Survey items

As part of a larger survey [17], we explored issues relevant to the diagnosis and management of NVAF as follows:

#### Risks assessment

GPs indicated the likelihood of performing nine tasks relevant to assessing stroke risk in a 68 year old regular patient without a history of prior stroke, newly diagnosed with NVAF after a resting electrocardiogram (ECG) (Table 2, additional file 1). The identification of hypertension, diabetes and congestive heart failure assists with the stratification of stroke risk in people affected by NVAF [1,5,21,22]. We therefore identified GPs who indicated that they would be "highly likely" to assess blood glucose levels, measure blood pressure and to carry out diagnostic cardiac procedures (transthoracic echocardiogram, chest x-ray or transoesophageal echocardiogram).

#### Estimate of stroke risk and the benefits of anticoagulation with warfarin

Three case scenarios were presented and GPs were asked to estimate the number out of 100 people identical to those described in each case who would experience a stroke in 12 months' time (Table 3, additional file 1). For each scenario, GPs indicated whether the "benefits of warfarin outweigh the risks", "the benefits and risks of warfarin are equally balanced", or "the risks of warfarin outweigh the benefits". We referred to clinical risk stratification schemes [1,5,21,22], including one local scheme [1], to classify the risk of stroke as "moderate-low" (case 1) and "high" (cases 2 and 3). Evidence-based guidelines advise that warfarin would optimise stroke risk reduction for all three cases, although aspirin may also be suitable for Case 1 [1].

#### Barriers

GPs were asked to consider seven reasons that might preclude anticoagulation for patients with NVAF. GPs were asked to estimate how often each reason applies to their patients when considering whether or not to prescribe warfarin. (Table 4, additional file 1).

#### Quality control of warfarin

GPs were presented with a case scenario of a "78 year old patient diagnosed with NVAF" after a stroke who "has recorded stable levels of INR within therapeutic range for the previous three months" on warfarin. GPs indicated how often they would measure INR levels and were asked to specify the target level. GPs then indicated the likelihood of enacting current guidelines for the management of supra-therapeutic INR levels [13]. GPs indicated the likelihood of ceasing "current warfarin therapy and resume at a lower dose when INR levels are within therapeutic range" if this patient recorded an INR level of 5.0 with no evidence of bleeding on 8 mg of warfarin per day. GPs were asked how many days after this consultation they would reassess the patient's INR. Existing national guidelines recommend reassessing INR levels within 24 hours of an abnormal result [13].

#### Strategies to assist in managing NVAF

GPs were asked to indicate the usefulness of strategies to assist in managing NVAF (Table 5, additional file 1). The strategies included practice resources (n = 6), training initiatives (n = 2), services (n = 5) and financial incentives (n = 2).

#### Satisfaction with services

GPs rated their satisfaction with their access to services for managing patients with stroke and at risk of stroke. (Table 4, additional file 1). Services included access to specialists (n = 3), hospital services (n = 4) and diagnostic tests (n = 4).

### Sample Size

We aimed to receive 600 questionnaires to estimate proportions with a confidence interval not exceeding ± 2% of the true value.

### Analysis

Predictors of outcomes were identified using multivariate logistic regression. Variables in Table 1 (additional file 1) were identified as potential predictors in our multivariate analyses and selected for screening to include in baseline multivariate models. GP risk assessment, GPs' assessments of whether the benefits of warfarin outweighed the risks for each of the three case scenarios, and GPs' self-reported quality control of warfarin were defined as outcomes. Potential predictors with a bivariate association with the outcome (p < 0.25) were entered into the model and the least significant variable was successively removed at each step until only significant predictors remained (p < 0.05) [23]. Therefore, the results present adjusted odds ratios in which the odds ratios were adjusted for the effects of all other variables from Table 1 (additional file 1) which remained significant (ie p < 0.05). All analyses were carried out using SPSS for Windows (version 15.0.1.1) [24].

### Ethics approval

The study protocol was approved by The University of New South Wales, Human Research Ethics Committee (HREC# 05087).

## Results

### Response

Seventy-four doctors were ineligible because they were not in general practice (n = 42), had retired (n = 11) or died (n = 1), were ill (n = 2), could not be contacted (n = 3), were on extended leave (n = 12) or had left Australia (n = 3). We received 596 questionnaires from 926 eligible GPs (64.4%). Respondents appeared representative of Australian GPs when compared with government statistics, although full-time GPs were over-represented (Table 1, additional file 1). All percentages reported here are based on a denominator of 596 unless otherwise indicated.

### Risk assessment

The majority of GPs stated that they would be "highly likely" to auscultate the heart (86.9%) and measure the blood pressure (90.9%) of a regular 68 year old patient newly diagnosed with NVAF. Just over half (54.2%) would be "highly likely" to determine blood glucose levels while 22.8% and 30.7% would be "highly likely" to refer the patient for a chest x-ray or transthoracic echocardiogram, respectively. Only 2.7% would be "highly likely" to refer the patient for a transoesophageal echocardiogram (TOE) (see Table 2, additional file 1). Just under one-third of GPs (32.4%) would be "highly likely" to refer the patient to undergo a cardiac diagnostic test (either chest x-ray or transthoracic echocardiogram or TOE), to assess blood glucose levels and measure blood pressure, which would potentially enable risk stratification of the patient. Further, GPs who performed these diagnostic tests were also significantly more likely to be "highly likely" to refer the newly diagnosed patient to a specialist (39.4% vs 25.6%) (χ^2^_(1) _= 11.69) (p = 0.001). The number of years GPs had been practising was the only demographic and practice characteristic that significantly predicted whether or not GPs carried out relevant testing for risk stratification. GPs who performed behaviours relevant for risk stratification had been in general practice for fewer years compared with those who did not (Mean = 17.8 years vs Mean = 19.6 years) (p = 0.046).

### Estimates of stroke risk and the benefits of warfarin

When asked to consider 100 patients identical to the 65 year old patient at moderate to low risk of stroke depicted in Case 1, the estimated median risk of stroke was 5% per year (IQR = 2.5–10). This estimated median risk was significantly lower than the estimated median risk for the 65 year old patient represented in Case 2 at high risk of stroke (Median = 10, IQR = 6–20) (p < 0.0001) and for the 75 year old "high risk" patient (case 3) (Median = 10, IQR = 5–20) (p < 0.0001). There was no significant difference in the median estimated risk of stroke between the patients presented in Cases 2 and 3, who were both at high risk of stroke (p = 0.09) (see Table 3, additional file 1).

Overall, 33.6% (n = 200) of GPs gave a correct estimate for the annual stroke risk for the 65-year old patient with a moderate to low risk of stroke presented in Case 1 (2–5% per year). Just under one-quarter (23.8%; n = 142) and 20.6% (n = 123) correctly estimated the annual stroke risk for the 65-year old high risk patient depicted in Case 2 and for the high risk 75-year old patient depicted in Case 3 (6–12%). Most commonly, GPs reported that they did not know the risk of stroke for each of the cases (n = 202, 33.9% for Case 1; n = 231, 38.8% for Case 2, and n = 232, 38.9% for Case 3). There was a tendency to be more likely to over-estimate rather than under-estimate the risk of stroke for Cases 1 and 2 (22.7% vs 9.4% for Case 1; 22.7% vs 14.4% for Case 2). A similar proportion over- and under-estimated the risk of stroke for Case 3 (21.8% vs 18.0%).

The majority of GPs indicated that the "the benefits of warfarin outweigh the risks" for the 65-year old patient depicted in Case 1 (n = 377, 63.3%) (Table 3, additional file 1), although a significantly greater proportion indicated that the "benefits of warfarin outweigh the risks" for the 65 year old patient at "high risk" of stroke depicted in Case 2 (63.3% vs 87.6%) (p < 0.001). Although the 75 year old patient depicted in Case 3 was also at high risk of stroke, GPs were no more likely to indicate that the "benefits of warfarin outweigh the risks" for this patient compared with the patient represented in Case 1 (moderate to low risk)(67.4% vs 63.3%) (p = 0.09), but were more likely to indicate that the benefits of warfarin outweigh the risks for the patient represented in Case 2 (87.6% vs 67.4%) (p < 0.0001).

### Predictors of GPs indicating that the "benefits of warfarin outweigh the risks"

Male GPs and those practicing in less socio-economically disadvantaged areas were significantly more likely to indicate that the benefits of warfarin outweigh the risks for the patient depicted in Case 1, at moderate to low risk of stroke (Adjusted Odds Ratio (AOR) = 1.80, 95% CI = 1.26–2.58, p = 0.001 and AOR = 1.08, 95% CI 1.02–1.15, p = 0.01, respectively). In addition, GPs with a full-time nurse employed in their practice were significantly less likely to indicate that the benefits of warfarin outweigh the risks, compared with those who employed a part-time nurse (AOR = 1.71, 95% CI 1.08–2.70) or those without a nurse attached to their practice (AOR = 1.51, 95% CI 1.01–2.24) (p = 0.03).

We did not identify predictors of indicating that the benefits of warfarin outweigh the risks for Case 2, as only 12.7% of GPs indicated otherwise in response to this scenario.

GPs who were male, those practising in less socio-economically disadvantaged areas and GPs who had participated in an education program about NVAF were all more likely to report that the benefits of warfarin outweighed the risks for the high risk patient presented in Case 3 (AOR = 1.47, 95% CI 1.02–2.12, p = 0.04; AOR = 1.09, 95% CI 1.03–1.16, p = 0.004 and AOR = 1.60, 95% CI = 1.03–2.48, p = 0.03, respectively).

### Quality control of warfarin

Most GPs stated that they would measure INR levels monthly (n = 463, 77.7%) for a 78 year old patient diagnosed with NVAF after a stroke who was currently receiving warfarin. Only 84 GPs (14.1%) would measure INR levels fortnightly. Almost 90% of GPs selected a target INR within the recommended range of 2.0–3.0 (n = 531, 89.1%) although 16.8% (n = 100) selected a target at the lower end of this range (2.0–2.5). A small minority selected a sub-therapeutic or supra-therapeutic target (n = 25, 4.2% and n = 34, 5.7%, respectively).

In response to an INR result of 5, 41.4% (n = 247) and 22.0% (n = 131) of GPs would be "highly likely" or "likely", respectively, to enact current guidelines for managing supra-therapeutic INR levels by ceasing warfarin and resuming the drug at a lower dose when INR levels became therapeutic [13]. However, 28.0% (n = 167) would be "highly unlikely" or "unlikely" to enact current guidelines in response to a supra-therapeutic INR.

Only 27.9% (n = 166) would reassess the patient's INR within one day after the consultation, as recommended by guidelines [13], while 32.2% (n = 192) of GPs would do so after two days and 39.1% (n = 233) would reassess INR levels three or more days after the consultation.

We classified GPs as adhering to evidence-based guidelines for the management of supra-therapeutic INR levels if they would be "highly likely" or "likely" to enact guidelines for an INR of five and would reassess INR levels within one day of the consultation. Only 21.8% of GPs (n = 126) adhered to evidence-based guidelines when judged against these criteria. Compared with GPs who worked full-time, GPs who worked part-time were significantly more likely to adhere to evidence-based guidelines (AOR = 1.62, 95% CI = 1.05–2.48) (p = 0.03). Fewer years in general practice (AOR = 0.96, 95% CI = 0.94–0.98) (p < 0.001) and practising in a less socio-economically disadvantaged suburb (AOR = 1.08, 95% CI = 1.01–1.16) (p = 0.03) were associated with an increased likelihood of adhering to evidence-based guidelines.

### Barriers to warfarin use

Contraindications to warfarin "usually" or "always" applied to the patients of 40.6% of GPs (n = 242) when considering whether or not to prescribe warfarin for their patients with NVAF. One-third of GPs (31.0%) indicated that this reason for not prescribing warfarin applied to their patients "sometimes". Just under one-third of GPs (32.7%) indicated that the "risk of adverse events will be unacceptably high" "usually" or "always" precluded anticoagulation, while 39.8% (n = 237) indicated that this reason "sometimes" applied to their patients.

Patients being at risk of falls was reported to "usually" or "always" apply to the patients of 24.0% of GPs when considering whether or not to prescribe warfarin, with 49.2% of GPs indicating that this "sometimes" applied to their patients.

Almost 60% (59.1%) of GPs reported that patient inability to comply with requirements for regular follow-up "never" or "rarely" applied to their patients, with only 10% endorsing this reason as "usually" or "always" applying to their patients when they considered whether or not to prescribe warfarin.

We had asked GPs to indicate how often patient reluctance to take warfarin applied to their patients when making decisions about warfarin. Just over two-thirds of GPs (69.8%) indicated that this reason for not prescribing warfarin applied to their patients at least "sometimes". Just over one-half of GPs (52.7%) stated that patient refusal precluded them prescribing warfarin at least "sometimes" (Table 4, additional file 1).

### Strategies for managing NVAF

Over 75% of GPs reported that patient education resources, point of care INR testing and a computerised risk calculator would be "quite" or "very useful" to assist in managing NVAF (Table 5, additional file 1).

A computerised practice NVAF register, a practice nurse and payments linked to preventive stroke management for NVAF were strategies endorsed as "quite" or "very useful" by 60% or more of GPs. An outreach specialist service for GPs to phone, fax or email questions and training to enable GPs to practice stroke prevention as a special interest were similarly popular. A centralised register for NVAF, anticoagulation clinics and pharmacist monitored INR levels were amongst strategies considered "not useful" or only "slightly useful" by more than 50% of GPs.

### Satisfaction with services for managing stroke and patients at risk of stroke

The majority of GPs were either "satisfied" or "highly satisfied" with their access to diagnostic services (see Table 4, additional file 1) with the exception of TOE (26.0%, n = 155). Compared with GPs located in capital cities or metropolitan areas, GPs located outside these areas were significantly less satisfied with their access to neurologists (50.7% vs 29.8%), cardiologists (80.1% vs 88.5%), emergency departments (65.5% vs 74.0%), stroke units (24.4% vs 40.4%), anticoagulation clinics (15.2% vs 26.4%), TOEs (19.5% vs 28.8%), and 24 hour holter monitoring (83.0% vs 89.9%) (p's < 0.001 to 0.04). However, these GPs were more likely to be "highly satisfied" or "satisfied" with their access to a medical bed for acute stroke in their local hospital (71.9% vs 56.0%) (p < 0.001).

## Discussion

This national, representative survey offers insights into how the evidence-based management of NVAF may be best implemented. These results have identified gaps in current practice that may be remedied by initiatives for implementing the evidence.

While the importance of assessing hypertension in patients with newly diagnosed NVAF was widely recognised, the significance of other risk factors was less recognised. While GPs could accurately determine that the risk of stroke was higher for the "high risk" scenarios (Cases 2 and 3) compared with the lower risk scenario (Case 1), GPs were no more likely to perceive that the benefits of warfarin outweighed the risks for the high risk 75 year old patient (case 3) compared with the lower risk 65 year old patient (case 1). This result suggests wariness to anti-coagulate aged patients, and demonstrates that concerns about anticoagulation are not limited to the very elderly (>80 years of age). Gaps in GPs' self-reported management of supra-therapeutic INR levels for patients using warfarin were identified suggesting that the communication of guidelines could be improved. More systematic dissemination and implementation of guidelines may be justified.

Our analysis has highlighted the challenges posed by Australian general practice. Access to specialists, particularly neurologists, does not appear to be satisfactory, and satisfaction appears to have decreased compared with an earlier survey [25]. Participation in educational programs about stroke risk and NVAF predicted appropriate appraisal of the risks and benefits of warfarin for the 75 year old patient at high risk of stroke, yet only 40% of GPs thus far had been able to access programs about stroke risk and only 25% had participated in programs about NVAF.

Caseload may also be important. Male GPs were more likely to identify when the benefits of warfarin outweighed the risks than female GPs. It is possible that female GPs have limited opportunity to manage patients with NVAF as they are more likely to practice part-time when compared with male GPs [19]. It was difficult to assess the independent effect of gender and part-time practice in our analysis because of the strong correlation between the two variables. Although NVAF accounts for a significant proportion of all strokes [2], the prevalence of NVAF is low [1] and the absolute numbers of patients with NVAF within individual practices may therefore be small. Therefore, male GPs may have greater experience in decision-making about NVAF, which may result in improved confidence about using warfarin. The survey may have also captured gender differences in risk seeking behaviour or risk aversion [26], although research on risk attitudes in physicians does not necessarily demonstrate gender differences [27,28]. As we did not directly assess these constructs we cannot speculate further on whether attitudes towards risk influenced our findings.

GPs whose practices were located in areas of greater socioeconomic disadvantage were less likely to endorse warfarin for patients either at "high" or "moderate to low risk" and were less likely to report behaviour indicating adherence to guidelines for managing supra-therapeutic INR levels. In Australia, residents in these areas are more likely to be born overseas and are less fluent in English than areas with less socioeconomic disadvantage [29]. This may limit GPs' ability to effectively engage their patients in discussions about the benefits and risks of warfarin for NVAF and reduce GP confidence in managing the condition. Moreover, residents in areas of greater socio-economic disadvantage have high rates of other stroke risk factors, such as hypertension, smoking, diabetes and obesity [30-33]. The management of these more prevalent conditions may displace the focus on NVAF.

The availability of a part-time nurse or no access to a practice nurse and fewer years in general practice were also associated with GPs' perceived benefits and risks of warfarin. The reason for this association is unclear, although practice nurses may be employed for specialist tasks. Therefore, this variable may be a proxy indicator for those GPs who have special interests that reflect particular strengths in clinical areas that are not relevant to stroke or aged care. In our survey, we found that a full-time nurse was more likely to be employed by GPs who indicated having a special interest in providing services for minor surgery (data not shown), giving some support for this interpretation.

In addition to fewer years in general practice, working part-time was also found to be associated with evidence-based self-reported management of supra-therapeutic INR levels. GPs who worked part-time were more likely to report behaviour indicating adherence to guidelines for managing supra-therapeutic INRs. These GPs may have less opportunity for following up patients and be more likely to institute immediate action because they may otherwise be unable to rectify supra-therapeutic INR levels. Alternatively, GPs who work part-time may be more likely to work in larger group practices and will therefore have the capacity to refer patients for follow-up compared with GPs who work full-time. GPs who work full-time may give consideration to providing continuity of care themselves, which may result in delaying the reassessment of INR levels. These explanations remain untested, however.

Fewer years in general practice predicted GP assessment of how the benefits and risks of warfarin are balanced as well as their self-reported management of supra-therapeutic INR levels. This result may reflect a cohort effect where more recently trained GPs may be more skilled in the principles of evidence-based medicine. This exposure during medical and post-graduate training may increase confidence in accessing and applying guidelines in their practice. However, we did not collect data to support this interpretation.

GPs indicated strategies that they would find most useful to assist them to manage NVAF. Practice tools and resources were favoured by the majority of GPs. These strategies can be easily incorporated within general practice and provide assistance with a broad spectrum of tasks ranging from risk assessment, patient tracking, treatment decision-making and the quality control of anticoagulation. Services that were located outside the practice were not favoured. The one exception was an outreach specialist service. GPs also favoured training sufficient to enable them to practice stroke medicine as a special interest, although special interest practices in stroke medicine do not currently exist in the Australian primary health care setting. An outreach service, if established, may be used to facilitate such training as well as improve access to specialists in stroke medicine.

Patient refusal or reluctance to take warfarin, was nominated to at least "sometimes" apply to the patients of more than half of the GPs surveyed. Other studies directly eliciting patient preferences have demonstrated that patient views about warfarin may indeed limit the wider uptake of warfarin [14]. Protheroe et al found that almost 40% of the 260 patients surveyed with atrial fibrillation aged 70–85 years preferred not to receive anticoagulation [34]. A recently reported randomised controlled trial compared guideline recommendations to a computerised decision-aid outlining the benefits and harms of warfarin individualised to a patient's own stroke risk [35]. One-quarter of patients receiving the decision-aid who were not already receiving warfarin started anticoagulation therapy, compared with 94% of those not already on warfarin receiving the evidence-based guidelines. It is important to note here that the existing literature on patient preferences is likely to be out of date as recent evidence from the BAFTA study [7] indicates the efficacy and safety of warfarin compared with aspirin. This new evidence may change the perceived benefit-harm trade-offs. Another study has found that patients themselves have a strong aversion to the name "warfarin"and are more likely to endorse warfarin when "blinded" to the name [15].

Even so, our results suggest that GPs often confront the real tension between a patient's preference to avoid anticoagulation, and perhaps their own willingness to prescribe warfarin to reduce the risk of fatal and disabling stroke. GPs themselves have indicated the importance of taking into account patient values [16]. Many GPs may be accepting of a patient's decision not to take warfarin, emphasising the importance of respecting patient values. Others have expressed concern that the way in which they framed the benefits and risks may have contributed to patients making "the wrong decision" [16]. While outcomes evaluating patient decision tools include knowledge and decisional conflict [36], the question of whether these tools reduce the experience of regret in the event of an "avoidable stroke" or "avoidable serious bleeding" remains unanswered.

More than 80% of GPs in this study nominated patient educational resources outlining the "pros and cons of available treatment" as either quite or very useful. While the evidence suggests that these resources may not promote sustained wider uptake of warfarin [36], these may nonetheless promote shared decision-making between GPs and their patients, improve patient understanding and assist with clarifying patient values.

## Limitations

While our relatively high response rate maximises the representativeness of findings, care in interpreting self-reported data is always required. Non-responders may have differed from responders in ways that we could not ascertain. Therefore, it is possible that our results would have differed if we had achieved a higher response rate. Moreover, our results may also be sensitive to the way in which we measured GP adherence to evidence-based guidelines and the criteria against which we judged GPs' responses. It must also be acknowledged that self-reported data may not faithfully reflect what GPs actually do when managing atrial fibrillation in their practice. Therefore, the difficulties in determining the validity of self-reported findings apply here. Further, these results may not be generalisable to other practising clinicians, such as cardiologists or neurologists, who are likely to manage relatively high numbers of patients with atrial fibrillation.

The survey itself was quantitative, and did not provide the opportunity for GPs to elaborate on their given responses. For example, the influence of case scenarios on decision-making was assessed using a quantitative approach which enabled us to determine how many GPs tip the balance in favour for or against warfarin. However, such an approach does not allow us to determine which aspects of the patient history GPs relied upon when assessing the clinical histories presented in the case scenarios. A qualitative approach would have also allowed greater latitude in introducing co-morbidities and other relevant aspects of a patient's medical history and invite discussion into how, for example, a history of bleeding or a history of falls would impact on decision-making.

Other issues relevant to GPs' adherence to guidelines were not assessed. Previous studies indicate that GPs may be sceptical of the evidence supporting the use of warfarin because of the select patient groups targeted in earlier randomised trials of warfarin [16]. While the results of the BAFTA study carried out in general practice with elderly patients (Mean age 81 years) had not yet been reported at the time of our survey [7], it remains to be seen whether the results of this trial will allay GPs' concerns about the limited generalisability of earlier studies. Other factors which could potentially influence warfarin uptake include the reluctance of GPs to challenge decisions made by medical specialists and concerns about liability should adverse events occur as a result of changing specialist recommendations [16]. Normative beliefs about what colleagues might or might not do may also be influential [16].

Our survey items for risk assessment and case scenarios were based on local evidence-based guidelines which provided estimates of actual stroke risk against which GP estimates could be compared. We acknowledge that other guidelines do not include TOE assessment for risk stratification and risk assessment [5,6]. However, transoesophageal findings, such as spontaneous echo contrast, have been shown to increase stroke risk [37], and were incorporated into guidelines that existed at the time our survey was conducted [1].

## Conclusion

Recent evidence identifies general practice as an appropriate and safe context in which to manage patients with NVAF [7]. This representative survey has alerted us to gaps in current care and GPs themselves have guided us on which implementation strategies are acceptable to reduce the risk of fatal and disabling strokes in people with NVAF.

## Competing interests

The authors declare that they have no competing interests.

## Authors' contributions

JW approached the other researchers with the idea of carrying out a research project about the management of NVAF in general practice. All authors contributed to the development of the questionnaire, questionnaire items and the interpretation of results. MG carried out the survey, analysed data and wrote the first draft of the manuscript. All others critically reviewed and edited the manuscript and MG revised the paper in response to feedback from other authors. All authors read and approved the final manuscript.

## Pre-publication history

The pre-publication history for this paper can be accessed here:



## Supplementary Material

Additional file 1 **Tables 1–5** Click here for file
