# Supplementary material for: The management of non-valvular atrial fibrillation (NVAF) in Australian general practice: bridging the evidence-practice gap. A national, representative postal survey
Source: BMC Fam Pract. 2008 Nov 13;9:62. doi: 10.1186/1471-2296-9-62 (PMC2611987; doi:10.1186/1471-2296-9-62)
Supplement: Additional file 1 — Tables 1–5 [file 1471-2296-9-62-S1.doc]

Table 1

| **Variable** | **Sample**  **N (%)**  **N=596*** | National Data **N (%)†** N=25,146 | p-value |
| --- | --- | --- | --- |
| *Age*‡ ≤44 years  ≥45 years | 206 (34.6)  390 (65.4) | 8,940 (35.6)  16,206 (64.4) | 0.60  0.60 |
| *Sex* Male  Female | 380 (63.8)  216 (36.2) | 15,745 (62.6) 9,401 (37.4) | 0.78  0.78 |
| Location Capital City/Metropolitan  Other | 418 (70.1)  171 (28.7) | 18,049 (71.8)  7,097 (28.2) | 0.37  0.79 |
| Work Hours Full-time  Part-time | 412 (69.1)  181 (30.4) | 14,789 (58.8)  10,357 (41.2) | <0.001  <0.001 |
| Type of practice Solo  Practise with you an other FP  Group practice | 78 (13.1)  81 (13.6)  434 (72.8) | NA | - |
| Years in family practice ≤19  20+ | 297 (49.8)  294 (49.3) | NA | - |
| ***Proportion of patients over the age of 65 years***  Median; IQR | 25 (15-40) | NA | - |
| ***Index of Relative Socio-economic Disadvantage of practice***  ***Median ranking (out of 10)* ‡‡**  Median; IQR  Member of:  Local Division  Royal Australian College of GPs (RACGP)  Fellow of RACGP  Australian Medical Association | 6 (4-8)  516 (86.6)  232 (38.9)  268 (45.0)  253 (42.4) | NA  NA | - |
| ***Employment of practice nurse***  Yes, full-time  Yes, part-time  No | 275 (46.1)  129 (21.6)  189 (31.7) | NA | - |
| Participation in:  Post-graduate training in family practice  EBM education program  Education program about stroke risk  Education program about NVAF | 308 (51.7)  450 (75.5)  242 (40.6)  139 (23.3) | NA | - |

*Percentages do not necessarily add to 100% due to missing data

**†**Australian Government Department of Health and Ageing statistics 2005/06^19^

**‡**Age at 1.01.06

**‡‡**Based on decile ranking of Index of Relative Socio-Economic Disadvantage scores assigned to postcodes. Decile rankings derived from 2006 Australian Census, based on postal code of GP practice (lower rankings indicate GP practice is located in areas of greater socio-economic disadvantage).^20^

N/A= not available

Table 2: GP self-reported likelihood of performing behaviours relevant to stroke risk assessment in a patient with newly diagnosed NVAF.

|  | Highly Unlikely | Unlikely | Neither unlikely  nor likely | Likely | Highly  Likely |
| --- | --- | --- | --- | --- | --- |
| Determine blood glucose levels | 5 (0.8) | 15 (2.5) | 55 (9.2) | 193 (32.4) | 323 (54.2) |
| Measure blood pressure | 5 (0.8) | 0 (0) | 1 (0.2) | 46 (7.7) | 542 (90.9) |
| Refer the patient for a chest x-ray | 5 (0.8) | 92 (15.4) | 152 (25.5) | 203 (34.1) | 136 (22.8) |
| Refer the patient for a  transthoracic echocardiogram | 30 (5) | 85 (14.3) | 104 (17.4) | 189 (31.7) | 183 (30.7) |
| Refer patient for a transoesophageal echocardiogram (TOE) | 190 (31.9) | 246 (41.3) | 100 (16.8) | 39 (6.5) | 16 (2.7) |
| Assess cardiac symptoms | 4 (0.7) | 0 (0) | 5 (0.8) | 120 (20.1) | 465 (78.0) |
| Auscultate the heart | 5 (0.8) | 2 (0.3) | 5 (0.8) | 62 (10.4) | 518 (86.9) |
| Classify the patient’s stroke risk as “low”, “medium” or “high” using standardised criteria | 30 (5) | 114 (19.1) | 119 (20.0) | 212 (35.6) | 117 (19.6) |
| Refer the patient to a specialist | 9 (1.5) | 68 (11.4) | 100 (16.8) | 237 (39.8) | 180 (30.2) |

Note: Percentages do not necessarily sum to 100 due to missing data

Table 3: Estimate of stroke risk and assessment of the benefits and risks of warfarin NVAF (N=596)*

| Case Scenario | GP estimate of stroke risk per year | Assessment of the benefits and risks of warfarin  N (%) |
| --- | --- | --- |
| Case 1: An otherwise healthy 65 year old patient diagnosed with NVAF. This patient does not have diabetes or hypertension. This patient does not have a history of stroke or cardiovascular disease. (Moderate-low risk) | Median (IQR): 5 (2.5-10)  Unsure 202 (33.9)  Actual risk 2-5% | Benefits of warfarin outweigh the risks 377 (63.3)  Benefits and risks equally balanced 56 (9.4)  Risks of warfarin outweigh the benefits 134 (22.5)  Unsure 28 (4.7) |
| Case 2: A 65-year old patient with NVAF. The patient has an abnormal echocardiogram demonstrating an enlarged left atrium with slow blood flow from the atrium (spontaneous echo contrast). This patient does not have a history of stroke or other cardiovascular disease. This patient does not have diabetes or hypertension. (High risk) | Median (IQR) 10 (6-20)  Unsure 231 (38.8)  Actual risk 6-12% | Benefits of warfarin outweigh the risks 522 (87.6)  Benefits and risks equally balanced 33 (5.5)  Risks of warfarin outweigh the benefits 24 (4.0)  Unsure 15 (0.3) |
| Case 3: A healthy 75 year old patient with NVAF. This patient has a history of hypertension. This patient does not have a history of diabetes. This patient does not have a history of stroke or cardiovascular disease. (High risk) | Median (IQR) 10 (5-20)  Unsure 232 (38.9)  Actual risk 6-12% | Benefits of warfarin outweigh the risks 402 (67.4)  Benefits and risks equally balanced 80 (13.4)  Risks of warfarin outweigh the benefits 84 (14.1)  Unsure 28 (4.7) |

Note: Percentages do not necessarily add to 100% due to missing data.

Estimates of actual risk based on Hankey [1].

Table 4 Barriers to anticoagulant prescribing and GP satisfaction with access to services for managing stroke and stroke risk (N=596)*

| **Barriers: How often each reason applies to your patients with NVAF when considering whether or not to prescribe warfarin** | **Never/Rarely**  **N (%)** | **Sometimes**  **% (n)** | **Usually/Always**  **% (n)** |  |
| --- | --- | --- | --- | --- |
| Patient reluctance to take warfarin | 173 (29.0) | 324 (54.4 ) | 92 (15.4) |  |
| Patient refusal to take warfarin | 276 (46.3) | 181 (30.4) | 133 (22.3) |  |
| Regular monitoring of INR levels will be too impractical or inconvenient for the patient | 352 (59.1) | 177 (29.7) | 60 (10.1) |  |
| Risk of adverse events will be unacceptably high | 157 (26.3) | 237 (39.8) | 195 (32.7) |  |
| You feel the patient would be unable to comply with requirements for regular follow-up | 212 (35.6) | 251 (42.1) | 127 (21.3) |  |
| That patient has contraindications to warfarin | 163 (27.3) | 185 (31.0) | 242 (40.6) |  |
| Patient risk of falls | 153 (25.7) | 293 (49.2) | 143 (24.0) |  |
| **Satisfaction with access to services for managing stroke and stroke risk** | **Highly satisfied/satisfied** | **Not sure** | **Dissatisfied/Highly dissatisfied** | **Have never used** |
| Neurologists | 264 (44.3) | 62 (10.4) | 256 (43.0) | 12 (2.0) |
| Cardiologists | 511 (85.7) | 23 (3.9) | 56 (9.4) | 4 (0.7) |
| General physicians | 356 (59.7) | 88 (14.8) | 125 (21.0) | 23 (3.9) |
| Emergency departments | 425 (71.3) | 65 (10.9) | 96 (16.1) | 7 (1.2) |
| Medical bed for acute stroke in local hospital | 361 (60.6) | 113 (19.0) | 98 (16.4) | 22 (3.7) |
| Multidisciplinary stroke unit or team | 210 (35.2) | 192 (32.2) | 112 (18.8) | 79 (13.3) |
| Anticoagulation clinics | 137 (23.0) | 201 (33.7) | 64 (10.7) | 192 (32.2) |
| Transthoracic echocardiogram | 441 (74.0) | 92 (15.4) | 37 (6.2) | 24 (4.0) |
| Transoesophageal echocardiogram | 155 (26.0) | 235 (39.4) | 56 (9.4) | 143 (24.0) |
| Carotid duplex | 556 (93.3) | 25 (4.2) | 8 (1.3) | 5 (0.8) |
| 24-hour holter ECG monitoring | 521 (87.4) | 34 (5.7) | 35 (5.9) | 4 (0.7) |

*Percentages do not necessarily add to 100% due to missing data

Table 5: Strategies to assist GPs to manage patients with NVAF (N=596)*

| **Strategy** | **Not useful** | **Slightly useful** | **Quite useful** | **Very useful** |
| --- | --- | --- | --- | --- |
| **Practice resources**  A centralised disease register for NVAF to monitor patient care and outcomes | 130 (21.8) | 210 (35.2) | 196 (32.9) | 57 (9.6) |
| A computerised register flagging patients with NVAF | 53 (8.9) | 134 (22.5) | 278 (46.6) | 126 (21.1) |
| Point of care INR testing to provide immediate INR blood test results during consultations | 35 (5.9) | 88 (14.8) | 190 (31.9) | 280 (47.0) |
| Patient educational resources outlining the pros and cons of available treatments | 9 (1.5) | 53 (8.9) | 264 (44.3) | 267 (44.8) |
| A computerised risk calculator to quantify the risk of stroke in patients with NVAF | 26 (4.4) | 115 (19.3) | 238 (39.9) | 212 (35.6) |
| A practice nurse to flag patients for follow-up, monitor INR levels and refer to GP for the management of out of range INRs | 69 (11.6) | 116 (19.5) | 212 (35.6) | 196 (32.9) |
| **Training initiatives** |  |  |  |  |
| One-to-one telephone sessions with GP peers trained by specialists in stroke medicine | 134 (22.5) | 206 (34.6) | 183 (30.7) | 67 (11.2) |
| Training for GPs in the prevention of stroke to enable practice as a special interest | 81 (13.6) | 142 (23.8) | 238 (39.9) | 131 (22.0) |
| **Services** |  |  |  |  |
| An outreach specialist service where you could email/fax/phone with specific questions | 50 (8.4) | 162 (27.2) | 230 (38.6) | 150 (25.2) |
| Anticoagulation clinics for monitoring patients on anticoagulants | 167 (28.0) | 165 (27.7) | 150 (25.2) | 111 (18.6) |
| Pharmacists monitoring INR levels and recommending dose  adjustments of warfarin as required | 421 (70.6) | 101 (16.9) | 55 (9.2) | 15 (2.5) |
| A system for dispensing warfarin which can tailor dosages to those required by patients | 148 (24.8) | 196 (32.9) | 189 (31.7) | 57 (9.6) |
| Pathology providers contacting patients with INR results  and recommending dose adjustments of warfarin as required | 227 (38.1) | 87 (14.6) | 114 (19.1) | 164 (27.5) |
| **Financial incentives** |  |  |  |  |
| Payment linked to preventive stroke management in patients with NVAF | 110 (18.5) | 114 (19.1) | 179 (30.0) | 190 (31.9) |
| Chronic Disease Management Medicare benefits Schedule Items relevant to NVAF | 109 (18.3) | 126 (21.1) | 210 (35.2) | 146 (24.5) |

* Percentages do not necessarily add to 100% due to missing data.
